# Audiological Outcomes, Tinnitus, Quality of Life, and Tumor Size After Stereotactic Radiosurgery for Vestibular Schwannoma: A Prospective Study

**DOI:** 10.7759/cureus.117976

**Published:** 2026-10-15

**Authors:** Murillo B Marra, Valeriana C Guimarães, Jordanna F Lousek, Jean Paiva, José Ricardo G Testa

**Affiliations:** 1 Department of Otorhinolaryngology, Paulista School of Medicine, Federal University of São Paulo, São Paulo, BRA; 2 Department of Otorhinolaryngology, Rehabilitation and Readaptation Center Dr. Henrique Santillo (CRER), Goiânia, BRA; 3 Otorhinolaryngology Residency Program, Hospital das Clínicas, Federal University of Goiás (UFG), Goiânia, BRA; 4 Graduate Program in Health Sciences, Federal University of Goiás (UFG), Goiânia, BRA; 5 Department of Radiation Oncology, Associação de Combate ao Câncer em Goiás (ACCG), Goiânia, BRA; 6 Department of Radiation Oncology, Centro Brasileiro de Radioterapia, Oncologia e Mastologia (CEBROM), Goiânia, BRA; 7 Department of Otolaryngology - Head and Neck Surgery, Federal University of São Paulo, São Paulo, BRA

**Keywords:** hearing, quality of life (qol), stereotactic radiotherapy, tinnitus, vestibular schwannoma

## Abstract

Objective: Vestibular schwannoma (VS) is a benign tumor that is often associated with hearing loss and tinnitus. Stereotactic radiosurgery (SRS) has been widely used to treat these tumors. However, hearing preservation following the procedure remains a challenge, particularly regarding the influence of the intracochlear radiation dose. The study's objective was to evaluate the effects of SRS on hearing function, perceived hearing handicap, tinnitus, quality of life, and tumor control in patients with VS, as well as to investigate the association between intracochlear radiation dose and audiological outcomes.

Methods: This prospective study included 30 patients who underwent SRS between 2017 and 2020. Pure-tone and speech audiometry, the Hearing Handicap Inventory for Adults (HHIA), the Tinnitus Handicap Inventory (THI), and quality-of-life assessments were performed before treatment and after a mean follow-up of 21 months (range, 3-48 months). Patients were stratified according to the intracochlear radiation dose received (≤5 Gy, >5 and <6 Gy, and ≥6 Gy). Statistical analyses included the Wilcoxon signed-rank test for continuous variables and two-sided exact McNemar tests for categorical outcomes, with Holm adjustment for multiple comparisons.

Results: Audiometric classifications showed a significant redistribution after SRS, with fewer patients in category B (p=0.031) and more in category C (p=0.044). No significant changes were observed in HHIA or World Health Organization Quality of Life Instrument - Short Form (WHOQOL-BREF) categories. THI showed an increase in the proportion of patients classified as having mild tinnitus and a reduction in those classified as having severe tinnitus (p=0.020 and p=0.031, respectively). Significant tumor size reduction was observed in patients with ≥24 months of follow-up (p=0.02). No significant association was detected between intracochlear radiation dose and the audiological outcomes evaluated.

Conclusion: Tumor size reduction was observed after ≥24 months of follow-up. Audiometric and tinnitus classifications showed significant changes in the overall sample, whereas subjective hearing handicap and quality of life did not. No significant association was detected between intracochlear radiation dose and the audiological outcomes evaluated. These findings should be interpreted cautiously given the small sample size.

## Introduction

Vestibular schwannoma (VS) is the most common benign tumor of the posterior fossa, accounting for approximately 80% of cerebellopontine angle tumors [1]. The most common initial symptoms of VS are hearing loss, often accompanied by ipsilateral tinnitus in 65% of patients [2], and balance disorders. Approximately 5% of patients may have normal hearing, particularly when the tumor is very small and confined to the cerebellopontine angle [3]. Three management strategies are currently available for VS: microsurgical resection, radiation (stereotactic radiosurgery (SRS) and fractionated stereotactic radiotherapy), and observation. Given its high rates of long-term tumor control and favorable safety profile, SRS has become a widely accepted treatment option for patients with small- and medium-sized tumors [4-6].

Hearing preservation after SRS varies substantially according to patient selection and duration of follow-up [7,8]. Recent evidence indicates that serviceable hearing may be preserved in approximately 71% of patients at two years after SRS, but this proportion decreases to approximately 59% at five years and 38% at 10 years [8]. Similarly, a recent comprehensive meta-analysis demonstrated that hearing preservation outcomes following radiosurgery vary according to the duration of follow-up, with age and radiation dose emerging as important prognostic factors [7]. These findings highlight the importance of considering follow-up duration when evaluating auditory outcomes after SRS [7-9].

The relationship between intracochlear radiation dose and auditory outcomes following SRS has been investigated, including studies demonstrating an association between higher cochlear doses and lower rates of hearing preservation, particularly in patients with better baseline hearing [10,11]. In patients undergoing SRS, cochlear doses exceeding 6 Gy have been associated with an increased risk of hearing loss [10]. Nevertheless, the influence of intracochlear radiation on auditory function remains controversial, as factors such as age and tumor volume also appear to have a significant impact on hearing outcomes, suggesting that this process is multifactorial in nature [11].

Given these considerations, the primary objective of this study was to evaluate changes in hearing function following SRS in patients with VS. The secondary objectives were to assess changes in perceived hearing handicap, tinnitus severity, quality of life, and tumor size after SRS, and to investigate the association between intracochlear radiation dose and audiological outcomes.

## Materials and methods

Study design

This was a prospective study conducted from January 2017 to June 2020. The study was approved by the Research Ethics Committee of the Escola Paulista de Medicina, Universidade Federal de São Paulo (No. 1.413.317), and was conducted in accordance with the Declaration of Helsinki and Brazilian regulations governing the ethical principles of medical research involving human subjects. Written informed consent was obtained from all participants. Sixty patient records were initially screened, and 56 patients underwent evaluation. Following the assessment, 30 patients who met the inclusion and exclusion criteria and provided informed consent were enrolled in the study, which was conducted in two phases. In the first phase, performed before SRS, participants underwent audiometric evaluation and completed questionnaires related to hearing perception, tinnitus, and quality of life. In the second phase, conducted at a mean follow-up of two years after SRS treatment, the same procedures were repeated to allow for longitudinal comparison. The post-SRS follow-up period ranged from three to 48 months, with a mean duration of 21 months. The heterogeneity of the follow-up period, including the presence of patients with relatively short follow-up, was considered when interpreting the longitudinal outcomes. A prespecified subgroup analysis was performed among patients with available follow-up of at least 24 months.

Participants

Participants were identified through an active review of medical records from patients diagnosed with VS who were scheduled to undergo SRS during the study period at the tertiary academic referral center, Hospital de Câncer Araújo Jorge, in Goiânia, GO, Brazil. Eligible participants were adults of either sex, aged 18 years or older, with a diagnosis of VS measuring ≤ 3.5 cm in diameter based on magnetic resonance imaging (MRI) of the temporal bones or skull, and who were scheduled to undergo SRS from January 2017 to June 2020. Individuals were excluded if they had previously undergone radiotherapy and/or microsurgery for VS, or if they were unable to complete any of the study assessments. In addition, patients who underwent any other type of VS treatment during the 24-month follow-up period were excluded from the study.

Assessment

Audiological evaluation was performed using pure-tone and speech audiometry (AD229b diagnostic audiometer, Interacoustics A/S, Middelfart, Denmark). Hearing was classified according to the American Academy of Otolaryngology - Head and Neck Surgery (AAO-HNS) criteria [12]. The pure-tone threshold was calculated as the average of the air-conduction thresholds at 0.5, 1, 2, and 3 kHz. Class A was defined as a pure-tone threshold ≤30 dB with speech discrimination ≥70%; Class B as >30 and ≤50 dB with speech discrimination ≥50%; Class C as >50 dB with speech discrimination ≥50%; and Class D as any pure-tone threshold with speech discrimination <50%.

To evaluate self-perceived hearing before and after SRS, the Brazilian Portuguese validated version of the Hearing Handicap Inventory for Adults (HHIA), originally developed by Newman et al. [13] and culturally adapted and validated for Brazilian Portuguese by Aiello et al. [14], was used. It consists of 25 questions divided into two subscales: social (12 questions) and emotional (13 questions). The answers are scored as follows: yes (four points), no (zero points), and sometimes (two points). In the present study, we considered the following for the statistical analysis: mild, moderate, and severe handicap.

To assess tinnitus-related handicap, the Brazilian Portuguese validated version of the Tinnitus Handicap Inventory (THI), originally developed by Newman et al. [15] and culturally adapted and validated for Brazilian Portuguese by Schmidt et al. [16], was used. The instrument consists of 25 questions, with total scores ranging from zero to 100. Responses are scored as “yes” (four points), “sometimes” (two points), and “no” (zero points), whereby higher scores indicate greater impact of tinnitus on quality of life. For statistical analysis, THI scores were categorized as follows: no tinnitus (zero points), mild handicap (1-25 points), moderate handicap (26-50 points), severe handicap (51-75 points), and catastrophic handicap (76-100 points).

Quality of life was assessed using the Brazilian Portuguese validated version of the World Health Organization Quality of Life Instrument - Short Form (WHOQOL-BREF), originally developed by the WHOQOL Group [17] and validated for use in Brazil by Fleck et al. [18]. The questionnaire consists of 26 items, including two general questions on overall quality of life and 24 questions distributed across four domains: physical health, psychological health, social relationships, and environment. Responses are based on a Likert scale evaluating intensity, capacity, frequency, and satisfaction. Domain scores are converted to a 0-100 scale, with higher scores indicating better quality of life. For statistical analysis, scores were categorized as follows: 76-100%, excellent quality of life; 51-75%, good quality of life; 26-50%, fair quality of life; and 0-25%, poor quality of life.

Stereotactic radiosurgery

Patients underwent SRS using a Novalis TX linear accelerator system (Varian Medical Systems, Palo Alto, CA, USA) integrated with the Brainlab stereotactic system (Munich, Germany), with a mean single-fraction dose of 12.5 Gy. Radiosurgical planning was performed by fusing previously acquired computed tomography (CT) and MRI scans, allowing accurate delineation of the tumor and adjacent critical structures, as well as calculation of the intracochlear radiation dose. Tumor size was assessed on MRI and recorded in centimeters (cm). The same radiologist performed tumor delineation on pre- and post-SRS imaging to ensure consistency in tumor size assessment. To define cochlear volume, seven to 12 consecutive axial slices (1 mm thick) were obtained, with a mean of nine slices per patient. These images were used for volumetric determination of the cochlea and subsequent dosimetric analysis. The intracochlear radiation dose recorded in each patient's treatment plan was obtained using the BrainSCAN v3.5 treatment planning system (BrainLAB, Heimstetten, Germany) [19]. For the exploratory analysis, patients were categorized according to the recorded intracochlear radiation dose into three ranges: ≤5 Gy, >5 and <6 Gy, and ≥6 Gy. These dose ranges were selected to explore the potential association between different levels of cochlear radiation exposure and audiological outcomes, based on previous literature reporting heterogeneous cochlear dose parameters and thresholds, including mean cochlear dose cutoffs ranging from 3 to 5 Gy [20].

Statistical analysis

Statistical analysis was performed using Microsoft Excel 365 (Microsoft Corporation, Redmond, Washington, United States) and MATLAB® version R2015a (MathWorks, Inc., Natick, MA, USA). Microsoft Excel 365 was used for data organization and reconstruction of category-specific 2 × 2 tables, whereas MATLAB® was used for the statistical calculations. The Kolmogorov-Smirnov test was used to assess the distribution of continuous variables. Continuous pre- and post-treatment measurements were compared using the Wilcoxon signed-rank test. Because the original subject-level paired data were unavailable, category-specific pre/post 2 × 2 tables were reconstructed from the reported marginal counts under a maximum-concordance assumption, corresponding to the largest feasible number of unchanged observations in each category. Each category was dichotomized as the category of interest versus all other categories. Before-versus-after differences were assessed using two-sided exact McNemar tests. Standardized McNemar residuals were used to assess the direction of change, and category-specific p-values were adjusted within each instrument using Holm’s method. The resulting category-specific p-values are approximate and conditional on the reconstructed paired distribution.

## Results

The sample consisted of 30 patients with VS, with a mean age of 53.8 years; 17 (56.6%) were female. The audiometric, self-perceived hearing, tinnitus, and quality-of-life findings are presented in Table 1. Of the 30 patients included in the study, 15 (50.0%) received an intracochlear radiation dose ≤5 Gy, seven (23.3%) received a dose >5 and <6 Gy, and eight (26.7%) received a dose ≥6 Gy. The post-radiosurgery follow-up period ranged from three to 48 months, with a mean follow-up duration of 21 months. A ≥24-month post-SRS follow-up analysis was performed in the 15 patients from the original cohort who had reached at least 24 months of follow-up at the time of analysis and for whom the corresponding audiological assessments were available. The remaining 15 patients had not yet reached 24 months of follow-up and were therefore not included in this subgroup analysis. Their audiological outcomes and otologic symptoms are presented in Table 2.

**Table 1:** Audiological evaluation, perceived hearing handicap, tinnitus impact, and quality of life before and after SRS (n=30). Values are presented as n (%). Category-specific p-values are Holm-adjusted, two-sided exact McNemar p-values derived from a maximum-concordance reconstruction of the reported marginal counts. These p-values are approximate and conditional on the reconstructed paired distribution. SRS: stereotactic radiosurgery; HHIA: Hearing Handicap Inventory for Adults; THI: Tinnitus Handicap Inventory; WHOQOL-BREF: World Health Organization Quality of Life Instrument - Short Form

	Before SRS n (%)	After SRS n (%)	p
Audiometry			
A	4 (13.33)	2 (6.67)	0.500
B	14 (46.67)	6 (20.00)	0.031
C	5 (16.67)	12 (40.00)	0.044
D	7 (23.33)	10 (33.33)	0.500
HHIA			
Mild	11 (36.67)	7 (23.33)	0.250
Moderate	9 (30.00)	8 (26.67)	1.000
Severe	10 (33.33)	15 (50.00)	0.188
THI			
No	3 (10.00)	4 (13.33)	1.000
Mild	7 (23.33)	16 (53.33)	0.020
Moderate	9 (30.00)	6 (20.00)	0.750
Severe	11 (36.67)	3 (10.00)	0.031
Catastrophic	0 (0.00)	1 (3.33)	1.000
WHOQOL-BREF			
Excellent	5 (16.67)	10 (33.33)	0.188
Good	11 (36.67)	14 (46.67)	0.500
Fair	13 (43.33)	6 (20.00)	0.062
Poor	1 (3.33)	0 (0.00)	1.000

**Table 2:** Audiological evaluation, perceived hearing handicap, tinnitus impact, and quality of life evaluated with ≥24 months of follow-up after SRS (n=15). Values are presented as n (%). Category-specific p-values are Holm-adjusted, two-sided exact McNemar p-values derived from a maximum-concordance reconstruction of the reported marginal counts. These p-values are approximate and conditional on the reconstructed paired distribution. SRS: stereotactic radiosurgery; HHIA: Hearing Handicap Inventory for Adults; THI: Tinnitus Handicap Inventory; WHOQOL-BREF: World Health Organization Quality of Life Instrument - Short Form

	Before SRS n (%)	After SRS n (%)	p
Audiometry			
A	3 (20.00)	2 (13.33)	1.000
B	9 (60.00)	2 (13.33)	0.062
C	1 (6.67)	7 (46.67)	0.094
D	2 (13.33)	4 (26.67)	1.000
HHIA			
Mild	7 (46.67)	3 (20.00)	0.375
Moderate	4 (26.67)	4 (26.67)	1.000
Severe	4 (26.67)	8 (53.33)	0.375
THI			
No	1 (6.67)	3 (20.00)	1.000
Mild	2 (13.33)	6 (40.00)	0.500
Moderate	5 (33.33)	4 (26.67)	1.000
Severe	7 (46.67)	1 (6.67)	0.156
Catastrophic	0 (0.00)	1 (6.67)	1.000
WHOQOL-BREF			
Excellent	2 (13.33)	5 (33.33)	0.998
Good	5 (33.33)	6 (40.00)	0.975
Fair	7 (46.67)	4 (26.67)	0.890
Poor	1 (6.67)	0 (0.00)	1.000

Table 3 presents a comparison of tumor size before and after SRS according to follow-up duration (<24 months and ≥24 months) and intracochlear radiation dose category.

**Table 3:** Comparison of tumor size before and after SRS according to follow-up duration and intracochlear radiation dose category. P-values were obtained using the Wilcoxon signed-rank test. SRS: stereotactic radiosurgery

	Before SRS Mean (SD)	After SRS Mean (SD)	p
Total sample (n=30)			
Tumor size (cm)	1.78 ± 0.91	1.70 ± 0.85	0.24
Longitudinal follow-up subsample (n=15)			
<24 months	1.84 ± 0.87	1.87 ± 0.94	0.64
≥24 months	1.72 ± 0.98	1.52 ± 0.75	0.02
Stratification by intracochlear radiation dose category (n=30)			
≤5 Gy (n=15)	2.08 ± 1.00	2.02 ± 0.98	0.49
>5 and <6 Gy (n=7)	1.82 ± 0.92	1.57 ± 0.63	0.12
≥6 Gy (n=8)	1.18 ± 0.34	1.21 ± 0.49	0.86

Tumor size varied across intracochlear radiation dose categories, with smaller tumors observed in patients receiving ≥6 Gy compared with those receiving ≤5 Gy. In this small cohort, no significant association was detected between intracochlear radiation dose category and the audiological outcomes evaluated. These findings should be interpreted cautiously given the small sample size and the imbalance in tumor size across dose categories. Pure-tone audiometry results and the HHIA questionnaire scores remained stable in the analyses performed.

## Discussion

The present study evaluated the effects of SRS on auditory function, otologic symptoms, quality of life, and tumor control in patients with VS. The main findings from the initial post-SRS assessment demonstrated a significant redistribution of audiometric classifications, characterized primarily by a reduction in the number of patients classified as category B and an increase in those classified as category C, as well as significant changes in the mild and severe tinnitus categories. No significant changes were observed in the HHIA or WHOQOL-BREF categories, either in the overall sample or in the subsample followed for ≥24 months. Furthermore, tumor reduction was observed only after 24 months of follow-up. In this small cohort, no significant association was detected between intracochlear radiation dose and the audiological outcomes evaluated. These findings should be interpreted cautiously given the small number of patients within each dose category and the exploratory nature of the dose analysis.

Audiometric analysis demonstrated a significant redistribution of hearing classifications following SRS, characterized primarily by a reduction in the proportion of patients classified as category B and an increase in those classified as category C. This pattern suggests a shift toward less favorable audiometric classifications in the overall sample. Because both categories require speech discrimination of ≥50%, the observed redistribution may reflect greater impairment of pure-tone hearing thresholds rather than a marked decline in speech discrimination. This interpretation is consistent with previous studies reporting deterioration in hearing thresholds following SRS, with greater deterioration associated with lower rates of hearing preservation [21,22]. Similarly, Graamans et al. [23] reported progressive deterioration in hearing thresholds without significant changes in speech discrimination, suggesting that changes in pure-tone sensitivity may occur without a corresponding deterioration in speech discrimination after SRS. However, no statistically significant changes were observed across audiometric categories in the subgroup with ≥24 months of follow-up, indicating that the redistribution observed in the overall sample should not be interpreted as evidence of progressive hearing deterioration over longer follow-up. Hearing preservation following SRS in patients with VS remains challenging due to multifactorial mechanisms related to both treatment and the natural course of the disease, including radiation-induced cochlear damage, microvascular compromise, cochlear nerve injury, transient tumor expansion, and dysfunction of the auditory pathways [24-26]. Hearing preservation rates vary according to follow-up duration and patient characteristics, with rates of 59.4% reported after a mean follow-up of 6.7 years [4], and rates of 86%, 82%, and 70% at one, two, and three years, respectively, described in patients with Koos grade I tumors [25]. More favorable outcomes have been associated with younger age, better baseline hearing, smaller tumor volume, and lower cochlear radiation doses, with hearing preservation rates reaching up to 92% under optimal conditions [4]. In contrast, cochlear doses exceeding 3 Gy and elevated intracanalicular pressure appear to increase the risk of hearing deterioration [11,24-26]. Furthermore, hearing decline has been observed independently of tumor growth and even in cases of spontaneous tumor regression, underscoring the complexity of the mechanisms involved in hearing loss among patients with VS [11,27].

Regarding subjective hearing handicap, no statistically significant changes were observed in the HHIA categories following SRS, either in the overall sample or in the subgroup with ≥24 months of follow-up. Notably, this finding occurred despite the significant redistribution observed in audiometric classifications, indicating that changes in objective hearing measures were not accompanied by a statistically significant change in perceived hearing handicap. This finding is consistent with a recent study of patients with VS treated with SRS, in which no statistically significant decline in Hearing Handicap Inventory scores was observed during long-term follow-up, despite changes in hearing preservation over time [28]. Previous research has shown that individuals with hearing loss may experience emotional, social, and communicative impairments, which may affect stress, anxiety, depression, and social isolation, particularly among older adults [29]. Furthermore, subjective perception of hearing handicap does not always correspond directly to objective audiological findings, and patients with VS may report functional impairment despite relatively preserved audiological performance [29]. In this context, subjective instruments such as the HHIA provide complementary information regarding the functional and psychosocial impact of hearing impairment that may not be fully captured by conventional audiological measures. Therefore, integrating subjective and objective assessments may contribute to a more comprehensive and patient-centered evaluation of patients with VS [30].

Regarding tinnitus, THI analysis demonstrated significant changes in the mild and severe categories following SRS, characterized by an increase in the proportion of patients classified as having mild tinnitus and a reduction in those classified as having severe tinnitus, whereas no statistically significant changes were observed in the no tinnitus, moderate, or catastrophic categories. Tinnitus is one of the most prevalent symptoms in patients with VS and has a multifactorial pathophysiology related to both cochlear impairment and neural dysfunction. Sensorineural hearing loss is considered an important contributor to the symptom and is often associated with recruitment and hyper-recruitment phenomena, as well as high-frequency auditory abnormalities [31]. However, central mechanisms also appear to be involved, as tinnitus may result from aberrant neural activity and alterations in cognitive and emotional networks, even in patients with normal hearing thresholds [31]. The redistribution toward lower tinnitus severity observed in the overall sample may therefore reflect the complex and multifactorial nature of tinnitus in patients with VS. Consistent with our findings, a recent meta-analysis including 7,281 patients reported a significant reduction in tinnitus after SRS (OR=0.63, p < 0.001), with a median follow-up of 75 months [32]. Nevertheless, the literature remains heterogeneous, with previous studies reporting improvement, stability, or worsening of tinnitus following radiosurgery, and differences in assessment methods and follow-up duration may contribute to these discrepant findings [33]. In the present study, no statistically significant changes were observed across THI categories in the subgroup with ≥24 months of follow-up, indicating that the redistribution observed in the overall cohort should be interpreted cautiously and should not be considered evidence of a sustained long-term improvement in tinnitus severity.

Quality of life assessment using the WHOQOL-BREF did not demonstrate statistically significant changes in any category following SRS in the overall sample. Although the distribution of patients across quality-of-life categories changed after treatment, with an increase in the proportion classified as having “good” or “excellent” quality of life and a reduction in those classified as having “fair” quality of life, these changes did not reach statistical significance. Similarly, no statistically significant changes were observed in any WHOQOL-BREF category in the subgroup followed for ≥24 months. These findings are consistent with the longitudinal study by Pruijn et al. [34], which evaluated health-related quality of life in 170 patients with VS treated with SRS and found no statistically significant or clinically relevant changes over time in Penn Acoustic Neuroma Quality-of-Life (PANQOL) or 36-Item Short-Form Survey (SF-36) physical and mental component scores. More recently, Siddiqui et al. evaluated 388 patients treated with Gamma Knife SRS and found no statistically significant decline in Hearing Handicap Inventory (HHI), Dizziness Handicap Inventory (DHI), THI, or SF-36 scores over up to seven years of follow-up, supporting the possibility that patient-reported quality-of-life measures may remain relatively stable after SRS despite changes in specific clinical outcomes [28]. The multidimensional nature of quality of life assessment may contribute to this finding, as auditory and otologic symptoms represent only some of the factors influencing patients’ perceived well-being. Previous research has shown that factors such as lack of energy, anxiety, headache, and balance problems are important determinants of physical and mental quality of life in patients with VS [35]. Therefore, changes in individual symptoms or functional domains do not necessarily translate into parallel changes in global quality-of-life measures. In the present study, the absence of statistically significant changes in WHOQOL-BREF scores should therefore be interpreted in the context of the multidimensional nature of quality of life, the relatively small sample size, and the categorical approach used for analysis.

Tumor size analysis showed no significant reduction in the overall sample or among patients with follow-up periods shorter than 24 months. However, in the longitudinal analysis of patients with follow-up of 24 months or longer, a significant reduction in tumor size was observed following SRS, corroborating the delayed nature of the tumor response to radiosurgery. The primary therapeutic goal of SRS is to achieve tumor stabilization, with high long-term local control rates reaching approximately 93% at five years [6], whereas volumetric reduction is typically observed only after prolonged follow-up. During the first months after treatment, radiation-induced inflammatory changes and edema may lead to a transient increase in tumor volume, a phenomenon known as pseudoprogression. This may occur either early or late after treatment and can make it difficult to distinguish true tumor progression from treatment-related changes [35]. These findings underscore the need for long-term follow-up and careful monitoring, particularly in larger tumors, which carry a higher risk of treatment failure and may initially appear to grow before stabilizing or regressing [36]. In addition, cystic tumors tend to show a more significant volumetric response to SRS, with significant tumor reduction observed in a large proportion of macrocystic cases [37].

Stratification of patients according to the intracochlear radiation dose did not reveal significant differences in the audiological outcomes evaluated before and after SRS. Both audiometric findings and HHIA scores remained stable across the different intracochlear dose levels analyzed. Several studies have investigated the potential influence of intracochlear radiation dose on hearing preservation following SRS. Importantly, the dosimetric parameters and thresholds used in previous studies have not been standardized. Chung et al. [10] found that patients exposed to minimum cochlear doses greater than 6 Gy had a significantly higher risk of hearing loss (OR 32; p=0.02). Similarly, Chang et al. [11] demonstrated that higher maximum cochlear doses were associated with more rapid hearing deterioration in a multicenter analysis of 611 patients. However, other factors, including age, tumor volume, and baseline hearing status, also appear to substantially influence functional outcomes, suggesting that the intracochlear dose alone may not be sufficient to explain hearing outcomes following SRS. Therefore, the influence of the intracochlear radiation dose on auditory outcomes remains controversial in the literature and likely reflects a multifactorial interaction among tumor characteristics, individual radiosensitivity, and treatment-related parameters.

This study has several limitations that should be considered. The small sample size and the heterogeneity of the follow-up period (three to 48 months) may limit the generalizability of the findings. In particular, shorter follow-up periods may be insufficient to capture the longer-term effects of SRS on hearing and tumor response. Furthermore, the absence of the original subject-level paired data required reconstruction of category-specific paired distributions from marginal counts, limiting the ability to perform a global paired ordinal analysis such as the McNemar-Bowker or marginal homogeneity test. Therefore, the category-specific p-values should be interpreted as approximate and conditional on the reconstructed paired distribution. In addition, the 24-month longitudinal analysis was performed in a smaller subsample, reducing the statistical power of the long-term comparisons. The study was conducted at a single tertiary referral center, which may also limit the external validity of the findings. The absence of an observation or control group precludes a complete distinction between the effects of SRS and the natural progression of VS. Furthermore, information regarding hearing aid use was not systematically collected, and vestibular symptoms and facial nerve function were not formally assessed, although these factors may influence functional outcomes and quality of life. Finally, the audiological assessments were not performed under blinded conditions, which may have introduced observer or measurement bias, particularly for subjective outcomes. In addition, a formal assessment of inter-observer variability or blinded independent reassessment of cochlear delineation was not performed, which may limit the assessment of dosimetric reliability. An additional limitation is the imbalance in tumor size across intracochlear radiation dose categories, with smaller tumors observed among patients receiving ≥6 Gy than among those receiving ≤5 Gy. Tumor size and anatomical characteristics, including the distance between the tumor and the cochlea, may influence radiation planning and cochlear exposure and therefore represent potential sources of confounding. Thus, the absence of a statistically significant association between intracochlear radiation dose and audiological outcomes in our cohort should be interpreted cautiously and does not exclude a clinically relevant effect of cochlear radiation dose. Future studies with larger samples should incorporate detailed volumetric dosimetric analyses, including mean cochlear dose (Dmean), maximum dose (Dmax), and cochlear volume receiving specific dose thresholds (e.g., V3Gy), as well as standardized assessments of vestibular and facial nerve function and hearing aid use.

## Conclusions

Following SRS, a significant reduction in tumor size was observed in patients with ≥24 months of follow-up. Audiometric analysis showed a significant redistribution of hearing classifications in the overall sample, characterized by a reduction in category B and an increase in category C, although no statistically significant changes were observed among patients followed for ≥24 months. Subjective hearing handicap measured by the HHIA did not show statistically significant changes following SRS, whereas THI analysis demonstrated a redistribution toward lower tinnitus severity in the overall sample, with an increase in the mild category and a reduction in the severe category; these changes were not statistically significant in the ≥24-month subgroup. Similarly, no statistically significant changes were observed in WHOQOL-BREF categories either in the overall sample or among patients with ≥24 months of follow-up. No statistically significant association was detected between intracochlear radiation dose and the audiological outcomes evaluated in this small cohort; however, this finding should be interpreted cautiously given the limited sample size and potential confounding related to tumor size and anatomical characteristics. Long-term audiological and patient-reported outcome monitoring, together with more detailed volumetric and dosimetric analyses, is warranted to further clarify the relationships between cochlear radiation exposure, hearing outcomes, tinnitus, and quality of life after SRS.
